# Mortality rate-dependent variations in the survival without major morbidities rate of extremely preterm infants

**DOI:** 10.1038/s41598-019-43879-z

**Published:** 2019-05-14

**Authors:** Jin Kyu Kim, Yun Sil Chang, Sein Sung, Won Soon Park

**Affiliations:** 1 Department of Pediatrics, Chonbuk National University School of Medicine, Jeonju, Korea; 2 Department of Pediatrics, Samsung Medical Center, Sungkyunkwan University School of Medicine, Seoul, Korea; 3 Research Institute of Clinical Medicine of Chonbuk National University-Biomedical Research Institute of Chonbuk National University Hospital, Jeonju, Korea

**Keywords:** Paediatrics, Paediatric research, Risk factors

## Abstract

The effects of improved survival of EPT infants on morbidity among survivors remain largely controversial. This retrospective cohort study of the Korean Neonatal Network data investigated whether the mortality rate of 23–24 weeks’ gestation was associated with survival without major morbidities in periviable 25–26 weeks’ gestation infants. The 2,083 eligible infants with 23–26 weeks’ gestation were grouped based on institutional mortality rate (group 1 and 2 ≤50% and >50% mortality rate, respectively, for 23–24 weeks’ gestation) and were further divided into 23–24 and 25–26 weeks’ gestation subgroups. The mortality rate of 23–24 weeks’ gestation infants was significantly lower in group 1 (32.7%) than in group 2 (69.9%). The survival without major morbidities rate for 25–26 weeks’ gestation infants was significantly higher in group 1 (31.2%) than in group 2 (18.5%). Antenatal steroid use and Apgar score at 5 min in group 1 were associated with decreased mortality and survival without major morbidities in 23–24 and 25–26 weeks’ gestation infants, respectively. In the multivariate analyses, decreased mortality rates in 23–24 weeks’ gestation infants were associated with higher survival without major morbidities rates in 25–26 weeks’ gestation infants due to decreased bronchopulmonary dysplasia, periventricular leukomalacia, and late-onset sepsis. Evidence-based perinatal and neonatal practices, including antenatal steroid use and better delivery room care contributing to decreased mortality in periviable 23–24 weeks’ gestation infants, were associated with lower morbidity and higher survival without major morbidities in more mature 25–26 weeks’ gestation infants.

## Introduction

Recent improvements in perinatal and neonatal intensive care have resulted in the improved survival of periviable, extremely preterm (EPT) infants^1–4^. However, EPT birth still remains a major cause of infant mortality and morbidity in survivors^5^. For this reason, there are concerns that the improved survival of EPT infants might be accompanied by increases in disabling morbidity in survivors^6,7^. In contrast, recent studies have reported that the improved survival of EPT infants has been accompanied by significantly increased survival without major morbidities, especially in more mature EPT infants^8,9^. Overall, the available data regarding the effects of the improved survival of EPT infants on morbidity among survivors remain largely controversial, and further studies are needed to clarify the issue.

In our previous single-centre studies, we observed that significantly improved survival of EPT infants with 23–24 weeks’ gestation was accompanied by a significantly reduced incidence of bronchopulmonary dysplasia (BPD) and nosocomial sepsis in more mature EPT infants with 25–26 weeks’ gestation^10,11^. However, as these studies reflect the experience of a single centre, the evidence is still very limited. Therefore, larger nationwide neonatal network data would be necessary to allow concrete conclusions to be drawn about the effects of the improved survival of EPT infants on major morbidity in survivors. The Korean Neonatal Network (KNN) is a nationwide, multicentre, prospective, web-based cohort registry system for very low birth weight infants (VLBWIs) with a birth weight <1,500 g^12^. In our previous study using the KNN data, we observed wide inter-hospital variations in the mortality rate of infants with 23–24 weeks’ gestation and evidence-based perinatal and neonatal management, including antenatal steroid use and better delivery room care, contributed to the enhanced survival of these EPT infants close to the limit of viability^13^. With the above in mind, we aimed to determine whether the improved survival of infants with 23–24 weeks’ gestation is associated with less major morbidity and more survival without major morbidities in more mature infants with 25–26 weeks’ gestation.

## Results

### Demographic and perinatal characteristics

According to the data of the National Statistical Office, the number of 23–26-week infants born in Korea in 2013–2016 was 3,005. In this study, 2,184 infants at 23–26 weeks were registered^14^. Table 1 shows a comparison of demographic and perinatal characteristics of the infants born at 23–24 weeks’ and 25–26 weeks’ gestation between the two study groups. In the overall group (infants with 23–26 weeks’ gestation), gestational age, birth weight, and caesarean section rate were significantly lower and antenatal steroid use, histologic chorioamnionitis, and *in vitro* fertilisation rates were significantly higher in group 1 compared to group 2. In infants with 23–24 weeks’ but not in those with 25–26 weeks’ gestation, the Apgar score at 1 and 5 min was significantly higher in group 1 than those in group 2. There were 68 births outside of hospital, 33/1074 (3.1%) babies were born outside of hospital in group 1 and 35/1009 (3.5%) babies were born outside of hospital in group 2. There were no significant differences in 23–24 weeks’ gestation in the 15/398 (3.8%) infants at 23–24 week’ gestation in group 1 and 9/322 (2.8%) infants in group 2.

**Table 1:** Comparison of perinatal and demographic characteristics between the two groups.

Characteristics	GA 23–24 W	n = 720	*p*-value	GA 25–26 W	n = 1,363	*p*-value	GA 23–26 W	n = 2,083	*p*-value
	Group 1 (n = 398)	Group 2 (n = 322)		Group 1 (n = 676)	Group 2 (n = 687)		Group 1 (n = 1,074)	Group 2 (n = 1,009)	
Gestational age (weeks)	23.6 ± 0.5	23.6 ± 0.5	0.793	25.5 ± 0.5	25.6 ± 0.5	0.127	24.8 ± 1.0	24.9 ± 1.0	0.007
Birth weight (g)	656.6 ± 107.5	656 ± 105.4	0.979	833.1 ± 160.5	843.3 ± 151.7	0.227	767.7 ± 166.6	783.8 ± 163.6	0.026
Apgar score, 1 min	2.9 ± 1.6	2.6 ± 1.6	0.025	3.7 ± 1.7	3.6 ± 1.8	0.891	3.4 ± 1.7	3.3 ± 1.8	0.435
Apgar score, 5 min	5.3 ± 2.0	4.9 ± 2.0	0.015	6.1 ± 1.8	6.0 ± 1.8	0.237	5.8 ± 1.9	5.7 ± 2.0	0.076
Hospital days (survival)	127.8 ± 40.4	133.8 ± 54.3	0.326	103.9 ± 37.1	106.5 ± 41.1	0.275	111.5 ± 39.7	111.2 ± 44.7	0.865
Body temperature (°C)	35.8 ± 0.9	35.6 ± 0.9	0.109	36.0 ± 0.6	36.0 ± 0.7	0.305	35.9 ± 0.8	35.9 ± 0.8	0.206
Male, n (%)	199 (50.0)	164 (50.9)	0.804	315 (46.6)	325 (47.3)	0.793	560 (52.1)	520 (51.5)	0.782
Antenatal steroid use, n (%)	323 (81.2)	214 (66.5)	<0.001	561 (83.0)	531 (77.3)	0.008	884 (82.3)	745 (73.8)	<0.001
Caesarean section, n (%)	235 (59.0)	196 (60.9)	0.620	482 (71.3)	524 (76.3)	0.037	717 (66.8)	720 (71.4)	0.023
PROM, n (%)	203 (51.0)	171 (53.1)	0.575	275 (40.7)	315 (45.9)	0.054	478 (44.5)	486 (48.2)	0.094
Histologic chorioamnionitis, n (%)	228 (57.3)	124 (38.5)	<0.001	308 (45.6)	234 (34.1)	<0.001	536 (49.9)	358 (35.5)	<0.001
HDP (%)	15 (3.8)	20 (6.2)	0.130	80 (11.8)	55 (8.0)	0.018	94 (8.8)	75 (7.4)	0.239
*In vitro* fertilisation, n (%)	115 (28.9)	79 (24.5)	0.190	166 (24.6)	122 (17.8)	0.002	281 (26.2)	201 (19.9)	<0.001

Values are expressed as mean ± standard deviation or number (%).

GA, gestational age; HDP, hypertensive disorder of pregnancy; PROM, premature rupture of membranes; W, weeks.

### Mortality and morbidity

Table 2 shows the mortality, composite morbidity, and survival without major morbidities of infants in the two study groups. The overall and gestational age-specific mortality rates in group 1 were all significantly lower than those in group 2. In the infants with 25–26 weeks’ but not 23–24 weeks’ gestation, the survival without major morbidities rate was significantly higher and the incidence of BPD and periventricular leukomalacia (PVL) were significantly lower in group 1 than in group 2. The overall incidence of late-onset sepsis (LOS) in the infants with 23–26 weeks’ gestation was significantly lower in group 1 than in group 2. The overall incidence of ROP in infants with 23–26 weeks’ gestation was significantly higher in group 1 than in group 2 (p = 0.001).

**Table 2:** Comparison of mortality, morbidity, and survival without major morbidities rate between the two groups.

(%)	GA 23–24 W	n = 720	*p*-value	GA 25–26 W	n = 1,363	*p*-value	GA 23–26 W	n = 2,083	*p*-value
	Group 1 (n = 398)	Group 2 (n = 322)		Group 1 (n = 676)	Group 2 (n = 687)		Group 1 (n = 1,074)	Group 2 (n = 1,009)	
Mortality*	130 (32.7)	225 (69.9)	<0.001	105 (15.5)	211 (30.7)	<0.001	235 (21.9)	436 (43.2)	<0.001
SWMM	19 (7.1)	8 (8.2)	0.709	178 (31.2)	88 (18.5)	<0.001	197 (23.5)	96 (16.8)	0.002
BPD	194 (72.4)	70 (72.2)	0.966	269 (47.1)	301 (63.2)	<0.001	463 (55.2)	371 (64.7)	<0.001
IVH	64 (23.9)	29 (29.9)	0.245	62 (10.9)	66 (13.9)	0.140	126 (15.0)	95 (16.6)	0.428
PVL	45 (16.8)	9 (9.3)	0.078	52 (9.1)	68 (14.3)	0.009	97 (11.6)	77 (13.4)	0.293
NEC	48 (17.9)	11 (11.3)	0.132	62 (10.9)	66 (13.9)	0.139	93 (11.1)	59 (10.3)	0.639
ROP	163 (60.8)	54 (55.7)	0.064	190 (33.3)	146 (30.7)	0.208	353 (42.1)	200 (34.9)	0.001
LOS	84 (31.3)	45 (46.4)	0.008	125 (21.9)	157 (33.0)	<0.001	209 (24.9)	202 (35.3)	<0.001

SWMM, survival without major morbidities at discharge; BPD, bronchopulmonary dysplasia ≥ moderate; GA, gestational age; IVH, intraventricular haemorrhage grade 3 or 4; LOS, late-onset neonatal sepsis; NEC, necrotizing enterocolitis stage ≥2; PVL, periventricular leukomalacia; ROP, retinopathy of prematurity stage ≥3; W: weeks.

*Percentages for morbidities refer to survival.

### Clinical factors associated with mortality and survival without major morbidities

Tables 3 and 4 shows the results of the multiple logistic regression analysis of the clinical factors associated with mortality and clinical factors associated with survival without major morbidities stratified by gestational age group, respectively. Adjusted variables were gestational age, birth weight (100 g increments), Apgar score at 5 min, antenatal steroid use, delivery mode, and histologic chorioamnionitis.

**Table 3:** Multiple logistic regression analysis of the clinical factors associated with mortality stratified by gestational age group.

GROUP 1	GA 23–24 W	*p*-value	GA 25–26 W	*p*-value	Total	*p*-value
	Adjusted OR (95% CI)		Adjusted OR (95% CI)		Adjusted OR (95% CI)	
Gestational age (weeks)	0.830 (0.519–1.329)	0.438	0.985 (0.647–1.499)	0.942	0.801 (0.662–0.970)	0.023
Birth weight (per 100 g)	0.751 (0.592–0.952)	0.018	0.841 (0.724–0.978)	0.024	0.805 (0.710–0.912)	0.001
Apgar score, 5 min	0.959 (0.850–1.081)	0.489	0.804 (0.711–0.908)	<0.001	0.876 (0.805–0.954)	0.002
Body temperature	0.780 (0.604–1.008)	0.057	0.752 (0.536–1.055)	0.099	0.770 (0.629–0.944)	0.012
Antenatal steroid use	0.548 (0.303–0.992)	0.047	0.879 (0.484–1.595)	0.671	0.702 (0.466–1.056)	0.089
Caesarean section	0.721 (0.450–1.154)	0.173	0.834 (0.504–1.380)	0.480	0.757 (0.540–1.062)	0.107
Chorioamnionitis	0.893 (0.559–1.426)	0.635	0.599 (0.373–0.959)	0.033	0.750 (0.541–1.038)	0.083
**GROUP 2**	**GA 23–24** **W**	***p*** **-value**	**GA 25–26** **W**	***p*** **-value**	**Total**	***p*** **-value**
Gestational age (weeks)	0.392 (0.216–0.711)	0.002	0.625 (0.450–0.867)	0.005	0.535 (0.446–0.643)	<0.001
Birth weight (per 100 g)	0.677 (0.501–0.914)	0.011	0.772 (0.679–0.877)	<0.001	0.757 (0.673–0.851)	<0.001
Apgar score, 5 min	1.015 (0.874–1.178)	0.848	0.984 (0.894–1.083)	0.737	0.988 (0.913–1.070)	0.769
Body temperature	0.926 (0.663–1.293)	0.651	0.961 (0.732–1.260)	0.772	0.936 (0.761–1.152)	0.535
Antenatal steroid use	1.168 (0.634–2.151)	0.618	0.750 (0.490–1.149)	0.186	0.850 (0.599–1.206)	0.362
Caesarean section	1.276 (0.727–2.238)	0.396	1.251 (0.816–1.919)	0.304	1.247 (0.891–1.746)	0.198
Chorioamnionitis	0.942 (0.543–1.636)	0.833	0.675 (0.463–0.985)	0.041	0.743 (0.547–1.008)	0.057
**TOTAL**	**GA 23–24** **W**	***p*** **-value**	**GA 25–26** **W**	***p*** **-value**	**Total**	***p*** **-value**
Gestational age (weeks)	0.661 (0.471–0.927)	0.016	0.777 (0.605–0.999)	0.049	0.682 (0.602–0.773)	<0.001
Birth weight (per 100 g)	0.757 (0.637–0.901)	0.002	0.827 (0.753–0.908)	<0.001	0.807 (0.743–0.876)	<0.001
Apgar score, 5 min	0.967 (0.888–1.054)	0.449	0.904 (0.841–0.973)	0.007	0.929 (0.878–0.982)	0.009
Body temperature	0.841 (0.693–1.019)	0.077	0.831 (0.676–1.021)	0.077	0.835 (0.726–0.961)	0.012
Antenatal steroid use	0.609 (0.409–0.906)	0.015	0.789 (0.564–1.103)	0.166	0.704 (0.547–0.906)	0.007
Caesarean section	1.011 (0.722–1.415)	0.951	1.108 (0.805–1.524)	0.529	1.045 (0.831–1.313)	0.708
Chorioamnionitis	0.687 (0.497–0.948)	0.022	0.604 (0.454–0.804)	0.001	0.639 (0.517–0.790)	<0.001

CI, confidence interval; GA, gestational age; OR, odds ratio; W, weeks.

**Table 4:** Multiple logistic regression analysis of the clinical factors associated with survival without major morbidities stratified by gestational age group.

GROUP 1	GA 23–24 W	*p*-value	GA 25–26 W	*p*-value	Total	*p*-value
	Adjusted OR (95% CI)		Adjusted OR (95% CI)		Adjusted OR (95% CI)	
Gestational age (weeks)	1.763 (0.651–4.774)	0.264	1.591 (1.136–2.229)	0.007	1.895 (1.511–2.375)	<0.001
Birth weight (per 100 g)	1.056 (0.676–1.649)	0.811	1.224 (1.078–1.389)	0.002	1.220 (1.080–1.378)	0.001
Apgar score, 5 min	1.275 (0.996–1.632)	0.054	1.292 (1.157–1.442)	<0.001	1.293 (1.169–1.431)	<0.001
Body temperature	1.070 (0.612–1.870)	0.813	1.041 (0.772–1.402)	0.793	1.047 (0.806–1.359)	0.733
Antenatal steroid use	1.023 (0.283–3.702)	0.793	1.016 (0.625–1.651)	0.950	1.001 (0.636–1.575)	0.996
Caesarean section	5.108 (1.474–17.706)	0.010	1.337 (0.899–1.987)	0.152	1.598 (1.106–2.308)	0.012
Chorioamnionitis	1.074 (0.462–2.501)	0.868	1.244 (0.871–1.776)	0.230	1.223 (0.880–1.699)	0.231
**GROUP 2**	**GA 23–24** **W**	***p*** **-value**	**GA 25–26** **W**	***p*** **-value**	**Total**	***p*** **-value**
Gestational age (weeks)	2.890 (0.740–11.279)	0.127	2.216 (1.424–3.448)	<0.001	1.945 (1.432–2.641)	<0.001
Birth weight (per 100 g)	1.263 (0.692–2.307)	0.447	1.195 (1.017–1.406)	0.031	1.201 (1.027–1.404)	0.022
Apgar score, 5 min	0.758 (0.548–1.047)	0.093	1.071 (0.947–1.210)	0.275	1.018 (0.911–1.138)	0.752
Body temperature	1.321 (0.604–2.889)	0.486	1.536 (1.066–2.212)	0.021	1.454 (1.044–2.027)	0.027
Antenatal steroid use	0.654 (0.187–2.282)	0.505	1.304 (0.745–2.280)	0.353	1.164 (0.703–1.925)	0.555
Caesarean section	0.813 (0.246–2.687)	0.734	1.010 (0.617–1.651	0.970	0.965 (0.614–1.516)	0.877
Chorioamnionitis	1.047 (0.325–3.369)	0.939	0.831 (0.527–1.312)	0.427	0.876 (0.575–1.334)	0.537
**TOTAL**	**GA 23–24** **W**	***p*** **-value**	**GA 25–26** **W**	***p*** **-value**	**Total**	***p*** **-value**
Gestational age (weeks)	2.019 (0.916–4.446)	0.081	1.739 (1.340–2.256)	<0.001	1.845 (1.545–2.204)	<0.001
Birth weight (per 100 g)	1.134 (0.796–1.616)	0.485	1.191 (1.081–1.312)	<0.001	1.190 (1.084–1.306)	<0.001
Apgar score, 5 min	1.070 (0.890–1.287)	0.470	1.194 (1.102–1.294)	<0.001	1.174 (1.091–1.264)	<0.001
Body temperature	1.107 (0.720–1.703)	0.643	1.223 (0.980–1.527)	0.075	1.193 (0.978–1.455)	0.081
Antenatal steroid use	0.831 (0.359–1.923)	0.665	1.184 (0.829–1.690)	0.353	1.122 (0.808–1.558)	0.490
Caesarean section	2.313 (1.025–5.218)	0.043	1.134 (0.839–1.533)	0.414	1.250 (0.945–1.654)	0.119
Chorioamnionitis	1.192 (0.611–2.326)	0.606	1.177 (0.899–1.542)	0.236	1.184 (0.923–1.521)	0.184

CI, confidence interval; GA, gestational age; OR, odds ratio; W, weeks.

In the infants with 23–24 weeks’ gestation, birth weight in both groups 1 and 2, antenatal steroid use in group 1 but not 2, and gestational age in group 2 but not 1 were clinical factors significantly associated with lower mortality (Table 3). In infants with 25–26 weeks’ gestation, birth weight and chorioamnionitis in groups 1 and 2 and Apgar score at 5 min in group 1 but not 2 were clinical factors associated with mortality.

In infants with 23–24 weeks’ gestation, caesarean section was associated with survival without major morbidities at discharge only in group 1 (Table 4). In the infants with 25–26 weeks’ gestation, gestational age and birth weight in both groups 1 and 2, Apgar score at 5 min in group 1 but not 2, and body temperature at admission in group 2 but not 1 were clinical factors significantly associated with survival without major morbidities.

Table 5 shows the adjusted odds ratios (ORs) and 95% confidence intervals (CIs) for mortality, survival without major morbidities at discharge, and each morbidity in group 1 versus group 2 with adjustment by multivariate logistic regression. In multiple logistic regression, significantly different clinical factors associated with mortality and survival without morbidities between group 1 and group 2 and previously known factors were adjusted. Therefore, we have chosen the clinical factors which were significantly different at least in 2 GA subgroups from Table 1. The adjusted variables were gestational age, birth weight (100 g increments), Apgar score at 5 min, antenatal steroid use, delivery mode, histologic chorioamnionitis, and *in vitro* fertilisation. In the infants with 23–24 weeks’ gestation, the OR of mortality was significantly increased and the incidence of PVL was significantly decreased in group 2 compared to group 1. In the infants with 25–26 weeks’ gestation, the ORs of mortality, BPD, intraventricular haemorrhage grade 3 or 4 (severe IVH), PVL, and LOS significantly increased and the OR of survival without major morbidities at discharge were significantly decreased in group 2 than in group 1.

**Table 5:** Adjusted odds ratios for mortality, morbidity, and survival without major morbidities according to group stratified by gestational age.

Subgroups	GA 23–24 W	*p*-value	GA 25–26 W	*p*-value	Total	*p*-value
	Adjusted OR (95% CI)		Adjusted OR (95% CI)		Adjusted OR* (95% CI)	
Mortality	5.276 (3.724–73475)	<0.001	2.456 (1.866–3.233)	<0.001	3.326 (2.684–4.120)	<0.001
Survival without major morbidities at discharge	0.613 (0.307–1.225)	0.166	0.421 (0.321–0.553)	<0.001	0.456 (0.354–0.587)	<0.001
BPD	1.165 (0.679–1.997)	0.579	2.244 (1.728–2.915)	<0.001	1.984 (1.566–2.512)	<0.001
IVH	1.385 (0.980–1.959)	0.065	1.483 (1.103–1.994)	0.009	1.444 (1.155–1.807)	0.001
PVL	0.522 (0.308–0.883)	0.015	1.610 (1.137–2.279)	0.007	1.121 (0.848–1.482)	0.423
NEC	0.672 (0.443–1.019)	0.062	1.277 (0.907–1.796)	0.161	0.992 (0.765–1.286)	0.950
ROP	0.729 (0.459–1.157)	0.179	0.924 (0.703–1.214)	0.571	0.876 (0.694–1.107)	0.267
LOS	1.091 (0.790–1.507)	0.596	1.407 (1.103–1.795)	0.006	1.268 (1.045–1.538)	0.016

BPD, bronchopulmonary dysplasia moderate or severe; CI, confidence interval; GA, gestational age; IVH, intraventricular haemorrhage grade 3 or 4; LOS, late-onset neonatal sepsis; NEC, necrotising enterocolitis stage ≥2; PVL, periventricular leukomalacia; ROP, retinopathy of prematurity stage 3 or 4; OR, odds ratio; W, weeks.

*The odds ratios correspond to group 2 vs. group 1. Values are adjusted by gestational age, birth weight per 100 g, Apgar score 5 min, antenatal steroid use, delivery mode, histologic chorioamnionitis, and *in vitro* fertilisation.

## Discussion

Besides improving mortality, increasing survival without major morbidities in periviable EPT infants is another important goal of neonatal intensive care. In a previous study, we reported that despite marked immaturity itself, the limit of viability in periviable EPT infants with 23–24 weeks’ gestation was not static and could thus be much improved by providing evidence-based perinatal and neonatal intensive care besides active treatment policies^10,11,15^. In the present study, the survival rate of EPT infants born at 23–26 weeks’ gestation was significantly higher in group 1 than in group 2, especially in the infants with 23–24 weeks’ gestation. This improved survival rate of EPT infants with 23–24 weeks’ gestation was accompanied by significantly lower morbidity (including BPD, severe IVH, PVL, and LOS) and resultant higher survival without major morbidities rates in more mature infants with 25–26 weeks’ gestation but not those with 23–24 weeks’ gestation. In concordance with our data, Yonge *et al*.^2^ reported that significantly reduced mortality in infants with 22–24 weeks’ gestation during the last decade in the NICHD NRN data was associated with a significantly improved rate of survival without neurodevelopmental impairment at 18–22 months of corrected age. Stoll *et al*.^1^ also showed that improved survival of infants with 23–24 weeks’ gestation during the last two decades in the NICHD NRN data was accompanied by survival without major morbidities in more mature infants with 25–28 weeks’ gestation. Overall, these findings suggest that evidence-based perinatal and neonatal clinical practices contributing to the decreased mortality rate of most immature infants born at 23–24 weeks’ gestation simultaneously contribute to improving survival without major morbidities in more mature EPT infants with 25–26 weeks’ gestation. As a result, considerably more EPT infants could enjoy potentially better clinical outcomes and quality of life^16^.

Identifying the clinical factors associated with mortality and survival without major morbidities is essential for developing new and effective clinical strategies, not only to overcome the limit of viability but also to improve the quality of life in periviable EPT infants^3^. In our previous studies, antenatal steroid use, Apgar score at 5 min, and body temperature at admission were significantly associated with decreased mortality in EPT infants^15,17^. In our previous study, Apgar score at 5 min and lower serum albumin level were the most effective universal predictors of mortality regardless of gestational age within postnatal day 7 (primarily due to pulmonary causes) and after postnatal day 7 (primarily due to capillary leak)^18^. In the present study, antenatal steroid use and Apgar score at 5 min in group 1 but not in group 2 were clinical factors associated with mortality in infants with 23–24 and 25–26 weeks’ gestation, respectively. For survival without major morbidities, Apgar score at 5 min in group 1 and body temperature at admission to the neonatal intensive care unit (NICU) in group 2 were clinical factors associated with survival without major morbidities in infants with 25–26 weeks’ gestation. Collectively, these findings suggest that applying evidence-based perinatal and neonatal intensive care practices, such as antenatal steroid use, better delivery room care including seamless resuscitation conducted by a well-experienced and skilful neonatologist, prevention of heat loss, better early admission care, and prevention of nosocomial sepsis, would be effective not only for improving the survival of periviable infants but also for simultaneously reducing major morbidities, thus increasing survival without major morbidities in the more mature EPT infants.

In the present study, significantly increased survival without major morbidities was achieved by significantly reduced morbidities (such as BPD, severe IVH, PVL, and LOS). Significantly reducing the incidence of severe IVH and PVL might contribute to reduced neurodevelopmental impairments^1,2^. In accordance with the present data, we previously observed that improved survival in infants with 23–24 weeks gestation was associated with the significantly reduced incidence of BPD and nosocomial sepsis^10,11^. However, in contrast, Stoll *et al*.^1^ reported that while major morbidities, including LOS, were significantly reduced for infants with 25–28 weeks’ gestation with improved survival of infants born at 23–24 weeks, the incidence of BPD was increased. Although few effective treatments are clinically available for treating BPD, wide variability in BPD occurrence across hospitals and some success in reducing the BPD incidence rate within individual hospitals through quality improvement efforts suggests that the identification and implementation of specific neonatal intensive care practices could modify the incidence of morbidities such as BPD^19,20^. In our previous studies, clinical implementation of a less invasive ventilation strategy, including early continuous positive airway pressure after early extubation and less use of oxygen, was effective for reducing not only LOS but also other major morbidities associated with extreme prematurity, such as BPD^10,11^. Taken together, these findings suggest that although it might be difficult to identify which specific clinical strategy plays a pivotal role, quality improvements through evidence-based perinatal and neonatal care practices as a whole can be quite effective for reducing the incidence of major morbidities, thereby increasing survival without major morbidities especially in the more mature infants with 25–26 weeks’ gestation, which is accompanied by the improved survival of the infants with 23–24 weeks’ gestation. Contrary to this, the overall incidence of ROP in infants with 23–26 weeks’ gestation was significantly higher in group 1 than in group 2. Increased incidence of ROP in the higher surviving group of VLBWI was due to competing outcomes, and further studies are necessary to clarify multiple factors associated with ROP.

The limitations of the present study include its retrospective and uncontrolled observational study design on prospectively collected nationwide cohort data. Furthermore, although our data are representative nationwide, including all tertiary NICUs in Korea, the temporal profile of the changes in the association between mortality and survival without major morbidities could not be analysed in this study because data were collected over a short time period. Lack of long-term follow-up outcome data is another limitation of this study. Although study group according to the mortality rate of infants with 23–24 weeks’ gestation was included as an independent factor in the multivariate analyses of associated clinical factors for mortality and survival without major morbidities, it is possible that unknown confounders might not have been controlled in this retrospective study. However, although the consistency of any specific clinical management policies within each group was not controlled, the significantly higher antenatal steroid use and Apgar score at 5 min in group 1 suggests that evidence-based perinatal and neonatal intensive care practices were provided in group 1 than in group 2. This implies that this nationwide study was well placed for identifying clinical strategies associated with decreased mortality and survival without major morbidities of periviable EPT infants, which might also redeem the limitations of the study.

In conclusion, a reduced mortality rate of infants born at 23–24 weeks’ gestation was accompanied by a significantly higher survival without major morbidities rate for the more mature infants with 25–26 weeks’ gestation, which was mostly attributable to decreased incidences of major morbidities, including BPD, severe IVH, PVL, and LOS. Antenatal steroid use and Apgar score at 5 min were clinical factors associated with decreased mortality and survival without major morbidities of EPT infants. Collectively, clinical implementation of more aggressive perinatal and improved neonatal intensive care practices could simultaneously lead to decreased mortality and survival without major morbidities in periviable EPT infants.

## Methods

### Patients

**Figure 1:**
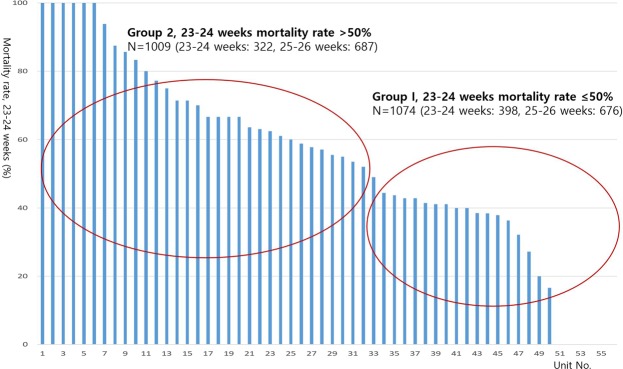
The wide variability of the mortality rate among infants born at 23–24 weeks gestation from the Korean Neonatal Network included in this study.

**Figure 2:**
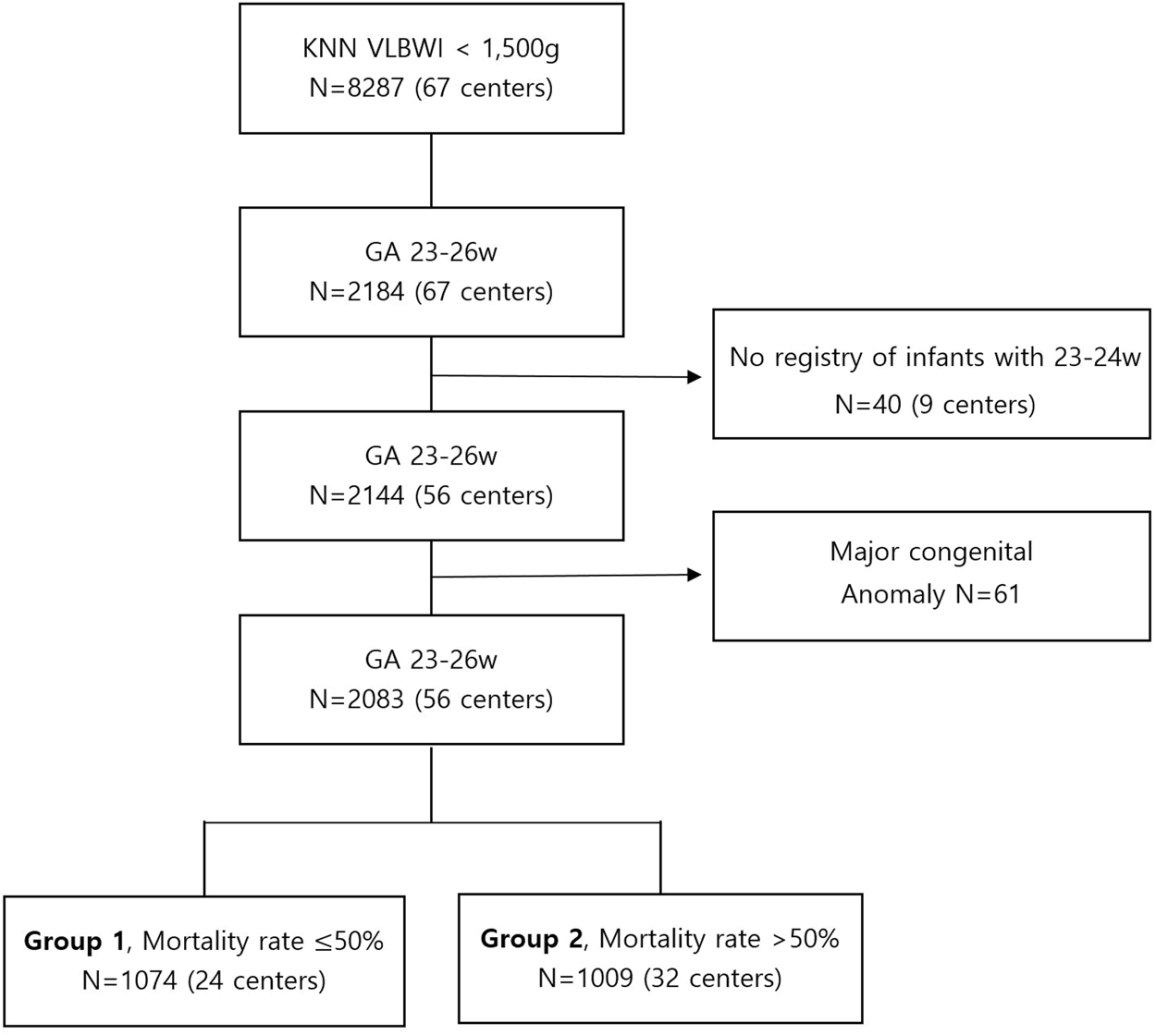
Study population from the Korean Neonatal Network database.

The database registry of the KNN prospectively registered the clinical information of VLBWIs admitted to the 67 voluntarily participating NICUs covering >80% of VLBWIs in Korea^12,21^. The enrolment criteria of KNN is registering only VLBWIs actively resuscitated in the delivery room, and admitted to the NICU in this study. Resuscitating infants >24 weeks’ gestation is mandatory by law in Korea, but most Korean tertiary NICUs are currently willing to resuscitate infants up to 23 weeks’ gestation. Trained staff used a standardised operating procedure to collect demographic and clinical information. Of the 8,287 VLBWIs born between January 1, 2013, and December 31, 2016, who were registered in the KNN, we collected data on 2,184 infants born at 23 weeks 0 days to 26 weeks 6 days of gestation. Forty ungrouped infants with 25–26 weeks’ gestation registered from the 9 NICUs were excluded as there was no registry of infants with 23–24 weeks’ gestation and we could not group these infants because of their lack of registry in the infants at 23–24 weeks’ gestation; moreover, 61 infants with major congenital anomalies were excluded from the study. Fifty-six of the 67 participating NICUs which had policy of actively resuscitating infant at 23–24 weeks enrolled more than one infant with 23–24 weeks’ gestation. We divided the units into two groups according to the (1) baseline mortality of the infants at 23–24 weeks’ gestation in this study and (2) the baseline mortality of 50% of the infants at 23–24 weeks’ gestation based on previous studies, which show that decreased mortality from 51% to 47% of infants at 23–24 weeks’ gestation is associated with increased survival without major morbidity of more mature infants at 25–28 weeks’ gestation (1). Given the wide institutional variation in the mortality rate of these infants, we divided all 2,083 infants with 23–26 weeks’ gestation into two groups: group 1 included patients from centres with a ≤50% mortality rate for 23–24 weeks’ gestation (1,074 patients from 24 hospitals) and group 2 included patients from centres with a >50% mortality rate for 23–24 weeks’ gestation (1,009 patients from 32 hospitals) (Figs 1 and 2). The infants were further stratified into those with 23–24 weeks’ gestation (n = 398 from 24 NICUs and 322 from 32 NICUs for groups 1 and 2, respectively) and 25–26 weeks’ gestation (n = 676 and 687 for groups 1 and 2, respectively).

We compared maternal and neonatal variables, including gestational age, birth weight, sex, antenatal corticosteroid use, chorioamnionitis, premature rupture of membranes (PROM), Apgar score at 1 and 5 min, hypertensive disorder of pregnancy, *in vitro* fertilisation, and body temperature at admission to the NICU, born inside or outside hospital between group 1 and 2 in the 23–24 and 25–26 weeks’ gestation subgroups.

We also compared mortality rates and various major morbidities, including BPD, severe IVH, PVL, necrotising enterocolitis (NEC), retinopathy of prematurity (ROP), and LOS between groups 1 and 2 in the 23–24 and 25–26 weeks’ gestation subgroups.

### Definitions

We complied a network database operation manual to define the patient characteristics. In the manual, gestational age was determined from the obstetric history based on the last menstrual period. Antenatal steroid use was defined as the administration of any corticosteroid to the mother at any time before delivery to accelerate foetal lung maturity. Chorioamnionitis was confirmed by placental pathology^22^ and PROM was defined as rupture of membranes over 24 hours before the onset of labour. BPD was defined as the use of supplemental oxygen or respiratory support at 36 weeks’ gestational age, corresponding to moderate to severe BPD using the severity-based definition for BPD of the National Institutes of Health consensus^23^. Severe IVH was defined as grade 3 or 4 according to the classification of Papile *et al*.^24^ PVL was defined as cystic PVL based on either head ultrasonography or cranial magnetic resonance imaging performed at ≥2 weeks of age. NEC was defined as ≥ stage 2b according to the modified Bell criteria^25^. ROP was defined as ≥stage 3 during the NICU admission according to an international committee for the classification of ROP^26^. LOS was defined by a positive blood culture obtained after postnatal day 7 in symptomatic infants suggestive of septicaemia and more than 5 days of antibiotic treatment. Survival without major morbidities was defined as survival without moderate to severe BPD, severe IVH, PVL, NEC, ROP, and LOS at the time of discharge.

### Statistical analysis

The characteristics of the study participants and their prenatal and neonatal morbidities were described as the mean ± standard deviation (SD) for continuous variables and as numbers and proportions for binary for categorical variables. Unadjusted comparisons between the two groups were performed using the chi-square or Fisher’s exact test for categorical data and the t-test for continuous data. Logistic regression was used to estimate the ORs with 95% CIs to identify the clinical factors between group 1 and group 2 associated with mortality and survival without major morbidities stratified by gestation age group. In multiple logistic regression, significantly different clinical factors associated with mortality and survival without major morbidities between group 1 and group 2 and previously known factors were adjusted. Models included covariates for gestational age, birth weight per 100 g, Apgar score 5 min, antenatal steroid use, delivery mode, histologic chorioamnionitis, and *in vitro* fertilisation. A *P*-value < 0.05 was considered statistically significant. Statistical analyses were performed using SPSS version 21 (IBM Corp., Chicago, IL, USA).

### Ethics statement

The KNN registry was approved by the institutional review board (IRB) at each participating hospital. Informed consent was obtained from the parents at enrollment by the NICUs participating in the KNN. Informed consent was waived by IRB for infants who died in the delivery room or at the early stage in the NICU before informed consent was able to be obtained for chart review. All methods were carried out in accordance with the IRB-approved protocol and in compliance with relevant guidelines and regulations.

## Data Availability

The datasets analysed during the current study are not publicly available due to the policy of Research of Korea Centers for Disease Control and Prevention but are available from the corresponding author on reasonable request.
